# Evaluation of Liver Function Tests to Predict Operative Risk in Liver Surgery

**DOI:** 10.1155/1995/47538

**Published:** 1995

**Authors:** Thomas Zoedler, Christoph Ebener, Heinz Becker, Hans D. Roeher

**Affiliations:** 1 Department of General Surgery Heinrich Heine University Düsseldorf Germany; 2 Klinik für Allgemein-und Unfallchirurgie Heinrich-Heine Universität Düsseldorf Moorenstr. 5 Düsseldorf 40225 Germany

## Abstract

Despite numerous studies in the past it is not possible yet to predict postoperative liver failure and safe limits for hepatectomy. In this study the following liver function tests ICG-ER (indocyaninegreen elimination rate), GEC (galactose elimination capacity) and MEGX-F (monoethylglycinexylidid formation) are examined with regard to loss of liver tissue and prediction of operative risk. Liver function tests were assessed in 20 patients prior to liver resection and on the 10th. postoperative day. Liver and tumor volume were measured by ultrasound and pathologic specimen and the parenchymal resection rate was calculated. In patients without cirrhosis (n = 10) ICG-ER and MEGX-F remained unchanged after resection, GEC was reduced but did not correspond to the resection rate. Patients with cirrhosis (n = 10) had a significantly lower ICG-ER and GEC before resection than patients without cirrhosis. After resection these tests were unchanged. Patients with liver related complications and cirrhosis (n = 5) had lower ICG-ER and GEC than patients with cirrhosis and no complications. In the postoperative course all liver function tests in these patients were significantly lower compared to preoperative results. Comparing liver function tests ICG serves best to indicate postoperative liver failure. Liver function tests do not correspond with loss of liver tissue.


*HPB Surgery*, 1995, Vol. 9, pp. 13–18
Reprints available directly from the publisher
Photocopying permitted by license only

© 1995 OPA (Overseas Publishers Association)
Amsterdam B.V. Published in The Netherlands
by Harwood Academic Publishers GmbH
Printed in Malaysia

### Evaluation of Liver Function Tests to Predict Operative Risk in Liver Surgery

THOMAS ZOEDLER, CHRISTOPH EBENER, HEINZ BECKER and HANS D. ROEHER

Department of General Surgery, Heinrich Heine University Düsseldorf, Germany

(*Received May 10, 1994*)

Despite numerous studies in the past it is not possible yet to predict postoperative liver failure and safe limits for hepatectomy. In this study the following liver function tests ICG-ER (indocyaninegreen elimination rate), GEC (galactose elimination capacity) and MEGX-F (monoethylglycinexylidid formation) are examined with regard to loss of liver tissue and prediction of operative risk. Liver function tests were assessed in 20 patients prior to liver resection and on the 10th. postoperative day. Liver and tumor volume were measured by ultrasound and pathologic specimen and the parenchymal resection rate was calculated. In patients without cirrhosis (n = 10) ICG-ER and MEGX-F remained unchanged after resection, GEC was reduced but did not correspond to the resection rate. Patients with cirrhosis (n = 10) had a significantly lower ICG-ER and GEC before resection than patients without cirrhosis. After resection these tests were unchanged. Patients with liver related complications and cirrhosis (n = 5) had lower ICG-ER and GEC than patients with cirrhosis and no complications. In the postoperative course all liver function tests in these patients were significantly lower compared to preoperative results. Comparing liver function tests ICG serves best to indicate postoperative liver failure. Liver function tests do not correspond with loss of liver tissue.

KEY WORDS: Liver function tests resection operative risk liver failure

### INTRODUCTION

Liver resection is the only method to cure primary and metastatic liver tumors. Standardization of the operation has led to a decrease in operative mortality, but the mortality rate is still near 20%. In approximately 70% HCC is combined with cirrhosis (Table 1). These persons bear a high risk of developing liver insufficiency, since liver function and liver regeneration is impaired[6,9]. Despite numerous studies in the past it is not yet possible to predict postoperative liver failure and safe limits for hepatectomy. Some authors prefer the determination of protein synthesis like coagulation analysis or urea-nitrogen-synthesis-rate[8] to estimate preoperative liver function in cirrhotic patients but a common standard does not exist yet. Quantitative liver function tests are well known in hepatology but since the test procedure is elaborate and therapeutic consequences are lacking their use is limited to special questions. Progress in surgery and transplantation induced a reestimation of these tests[3,4,10].

The tests commonly used now and employed in our study are the ICG, GEC and MEGX tests.

ICG is a dye which is excreted by the liver dependant on liver blood flow. The elimination rate normally amounts 16–20 %/min[11]. Galactose is converted to galactose-1-phosphate by the enzyme galactokinase. The elimination capacity should reflect the metabolically active liver tissue. The normal value is about 6, 5 mg/min kg[12]. MEGX is a metabolite of lidocaine.

*Address for correspondence*: H. D. Röher, Klinik für Allgemein-und Unfallchirurgie; Heinrich-Heine Universität Düsseldorf; Moorenstr. 5; 40225 Düsseldorf; Germany.

**Table 1** Review of the literature (reference)

| | *HCC* | *Cirrhosis* | *Mortality Rate* |
|---|---|---|---|
| Okamoto 1984[1] | 38 | 78% | 26% |
| Nagorny 1989[2] | 110 | 24% | 9% |
| Takenaka 1990[3] | 191 | 66% | 12,6% |
| Tsuzuki 1990[4] | 119 | 67% | 15,1% |
| Choi 1990[5] | 174 | 67% | 13,2% |
| Bismuth 1986[6] | 35 | 100% | 14% |
| Franco 1990[7] | 72 | 100% | 6,9% |
| Paquet 1991[8] | 23 | 100% | 13% |

The MEGX—formation is controlled by the cytochrome P 450 system in the liver and is supposed to indicate the active liver tissue as well[10].

In our study we raised the following issues:

1. Are the tests capable of recognizing a loss of liver tissue by hepatectomy since they are called "quantitative"?
2. Can they discreminate cirrhotic from unaffected livers?
3. Can we predict the operative risk of hepatectomy with the help of liver function tests?

### Methods

In 34 patients undergoing surgery for hepatectomy, cholecystectomy or hydatid cyst resection liver function tests were assessed prior to operation and on the 10th. postoperative day. Liver volume and tumor volume were determined by ultrasound at the same time in order to calculate the parenchymal resection rate described by Okamoto[1]. The resected liver specimen served as a control for the calculation of the volume resected. The incidence of cirrhosis was documented. The postoperative course was recorded with special referrence to signs of liver insufficiency like intractable ascites, coma or deficiant protein synthesis.

Liver resection was performed in 20 patients, 6 of them suffered from HCC. 10 Patients had liver cirrhosis proven by histological examination. Patients with gall stone disease and hydatid cysts served as a control group to show the effect of anaesthesia and operation trauma on liver function. Informed consent was obtained from each patient.

**Table 2** Study design

| | |
|---|---|
| 34 Patients | |
| Preoperative | • Liver function test<br>• Evaluation of liver volume and tumor volume |
| Operation | • Determination of resection volume |
| 10th. Day | • Liver function test<br>• Evaluation of residual volume |
| Postoperative | • Hospital morbidity and mortality |

**Table 3** Patients

| *Operation* | *n* | *Indication* | *n* | *Cirrhosis* |
|---|---|---|---|---|
| Liver resection | 20 | HCC | 6 | 6 |
| | | Metastasis | 14 | 4 |
| Pericystectomy | 7 | Hydatid cysts | 7 | 0 |
| Cholecystectomy | 7 | Cholelithiasis | 7 | 1 |

### Liver Function Tests

The ICG test was performed by the bolus injection technique described by Paumgartner[11]. The extinction of the dye was measured in 7 serum samples, 3 to 21 min after injection of 0,5 mg/kg ICG. The elimination rate was calculated by least square log linear-regression analysis of the descending portion of ICG extinction as a function of time.

The GEC-test was determined from serum samples drawn 0, 20, 25, 30, 35, 40, 45, 50 and urine sample 120 min after iv. injection of 0,5 g/kg galactose. The elimination capacity was calculated as proposed by Tygstrup[12].

The MEGX serum concentration was measured 15 min after iv. injection of 0,5 mg/kg lidocaine by fluoro polarization immuno assay (Abbott Laboratories, Chicago) specified by Oellerich[10].

Liver and tumor volume was determined pre- and postoperatively by ultrasound[13]. The parenchymal resection rate was calculated according to the method by Okamoto[1].

Supposing a normal distribution of the test values the student-t-test was applied to calculate the significance from mean values and standard deviation.

### RESULTS

No operative death occurred in 20 cases of liver resection but there were 5 late complications and 3 patients died because of liver failure. The majority of these complications was induced by preexisting liver cirrhosis. It seems that the extent of resection is not only necessarily a cause of liver failure. This may focus our interest to the question whether the degree of cirrhosis and consequently the risk of postoperative liver insufficiency can be determined prior to operation. Therefore we allocated our patients to 3 categories:

**Table 4** Hospital morbidity

| Pat. No. | Indication | Cirrhosis | Resection rate | Compli-cation | Death |
|---|---|---|---|---|---|
| 1 | HCC | Yes | 54% | Coma | 26th. day |
| 8 | Metastases | No | 40% | Ascites | No |
| 20 | HCC | Yes | 5% | Coma | 48th. day |
| 24 | HCC | Yes | 41% | Coma | 63th. day |
| 25 | HCC | Yes | 18% | Ascites | No |

Resections with unaffected livers; resections in patients with cirrhosis and uneventful postoperative course; resections in patients with cirrhosis and liver failure.

The results are shown separately for each test employed. (Fig. 1) The ICG-elimination rate apparently is not influenced by liver resection in normal livers and cirrhotic livers. Postoperative complications lead to a significant decrease of the elimination rate ($p = 0{,}004$ vs. preop in the same group). The difference between the preoperative values of noncirrhotic and cirrhotic livers is significant ($p = 0{,}001$). There is a further drop in the preoperative elimination rate of patients who will develop liver failure but this is not significant.

The galactose elimination capacity (Fig. 2) shows a significant decrease after resection in normal livers ($p = 0{,}003$). The regression equation however fails to show a correlation between the resection rate and the decrease of the elimination capacity ($r = 0{,}452174$). The difference between cirrhotic and non cirrhotic livers is significant ($p = 0{,}001$). Similar to the ICG elimination there is a further decrease of GEC in patients with liver failure. It cannot be explained why GEC remains unchanged after resection in cirrhotic patients. Postoperative complications result in a significant decrease of the GEC ($p = 0{,}036$ vs. preop).

Regarding the MEGX test (Fig. 3) we find a postoperative rise mainly in the control group and with decreasing extent in the resection groups. Only persons with complicated postoperative course have a lower MEGX value 15 min after lidocaine injection.

### DISCUSSION

Generally the operative risk in liver surgery is estimated on the basis of serum chemical liver profile and coagulation studies. Under the compensatory condition of the diseased liver these data are usually within or near normal limits. Therefore it is difficult to predict liver function following hepatectomy preoperatively. In consequence it is desirable to apply a test which is

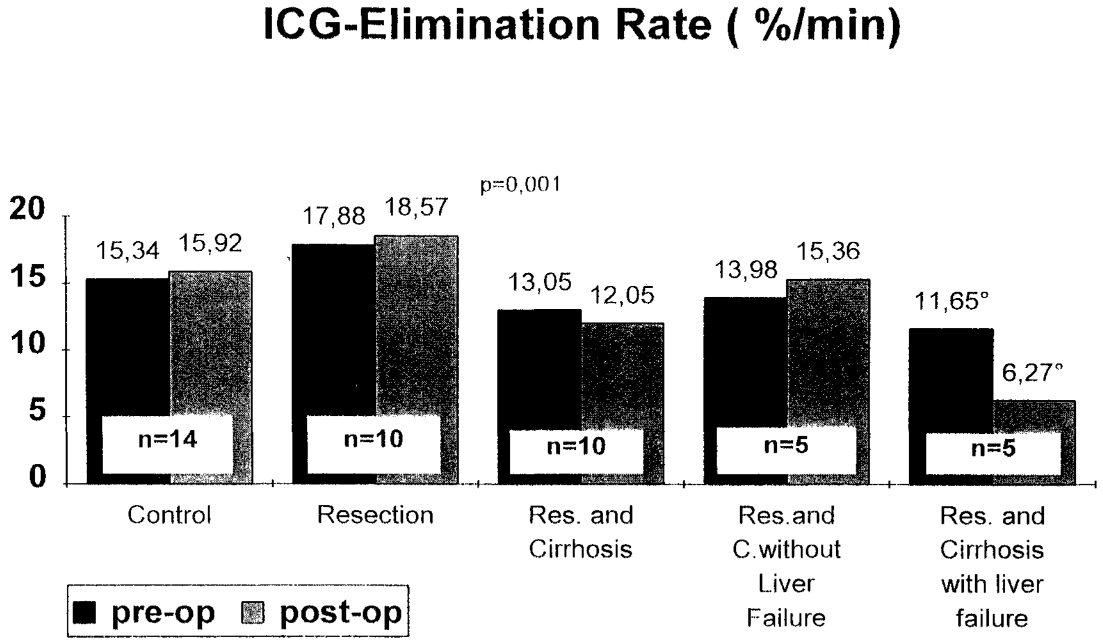

**Figure 1** Indocyanine Green elimination rate before and after cholecystectomy, liver resection in unaffected livers and cirrhotic livers. Patients with cirrhosis are allocated to a group with uneventful postoperative course or to a group with liver failure. $p = 0{,}001$ for ICG elimination rate in patients with unaffected livers versus patients with cirrhotic livers. °: $p = 0{,}004$.

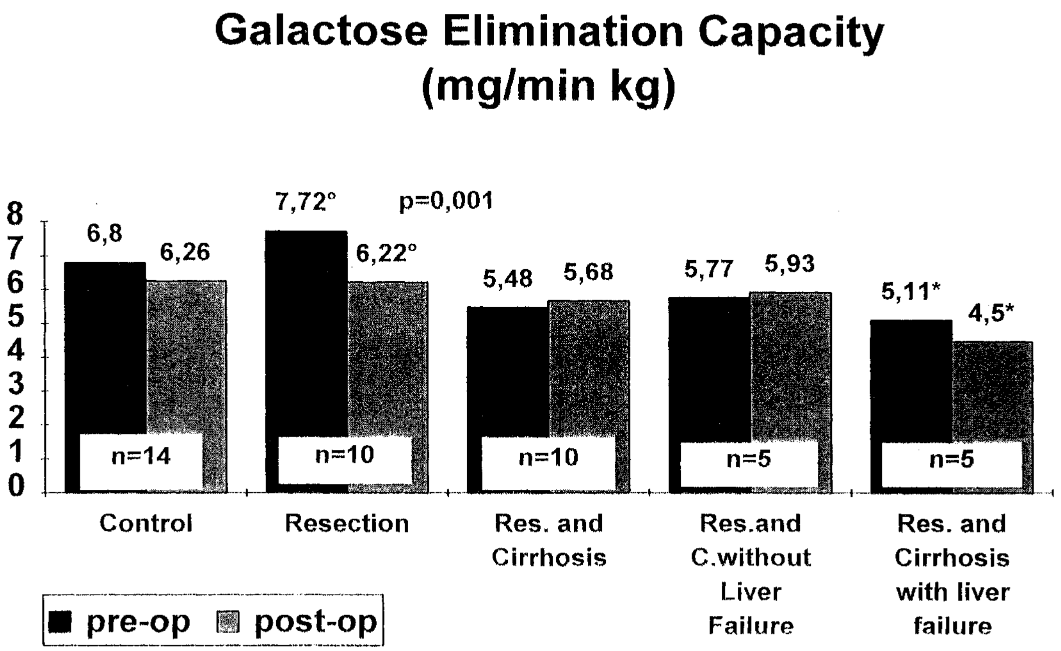

**Figure 2** Galactose elimination capacity before and after cholecystectomy, liver resection in unaffected livers and cirrhotic livers. Patients with cirrhosis are allocated to a group with uneventful postoperative course to a group with liver failure. The difference of GEC between patients with resection in unaffected livers and resection in cirrhotic livers is significant (p = 0,001). ° : p = 0,003; * : p = 0,036.

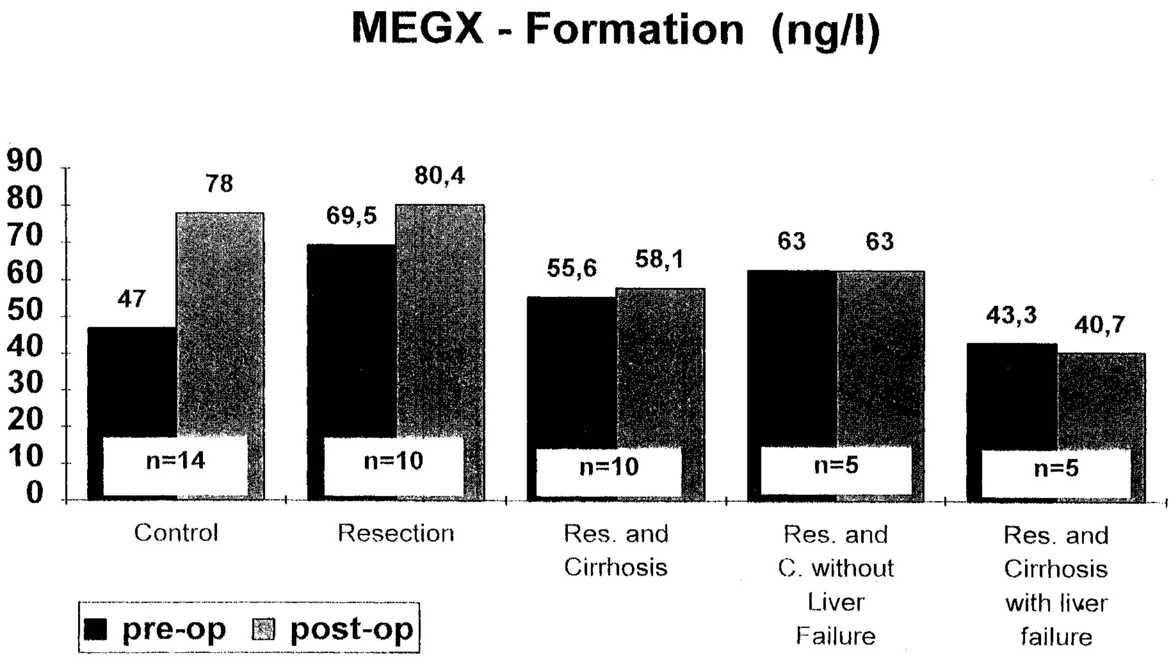

**Figure 3** Monoethylglycinexylidide formation before and after cholecystectomy, liver resection in unaffected livers and cirrhotic livers. Patients with cirrhosis are allocated to a group with uneventful postoperative course or to a group with liver failure.

capable of determining liver function and to estimate the limit of safe hepatectomy in those patients. Quantitative liver function tests are supposed to indicate the metabolically active liver volume and liver function. Several studies affirmed this statement. ICG is reduced after functional hepatectomy in dogs and induction of cirrhosis[14]. A positive correlation between liver volume and GEC has been found in aging man[15]. In our study only GEC indicates loss of liver tissue but the decrease of test level does not correlate with the resection rate. It has been shown that liver resection induces an additional capacity for metabolizing galactose[16]. This may serve as an explanation for our results, that liver resection is not followed by an adequate reduction of elimination capacity.

The ICG elimination rate performed by the bolus injection technique reflects the liver perfusion[11] and depends on intrahepatic shunting to be found in cirrhotic and tumor bearing livers[17]. Therefore it is not a valuable parameter of the active liver volume. As we know other application techniques are apt to show a volume dependant ICG elimination[18] but application form and calculation are complicated and not suitable for clinical use. The MEGX formation interferes with many drugs[19] and maintains a wide interindividual range of test values. In our opinion the test procedure which consists of only one blood sample after 15, 30 or 60 minutes, is not sufficient for analyzing liver function.

The value of the ICG test, GEC and MEGX test in indicating presence of cirrhosis is not doubted[10,20,21]. Since we are aware of liver cirrhosis by routine histological examination done preoperatively the superiority of the tests to simple methods like determination of the Child index is questioned[22,23]. The results of our examination present a significant difference between test values of cirrhotic and non cirrhotic livers only for ICG and GEC. In contrast to ICG and GEC the MEGX test fails to recognize cirrhotic livers.

The ICG elimination is diminished postoperatively in patients suffering from liver related complications[24]. This statement is confirmed by our results. Further information is awaited to select patients with impending liver insufficiency by preoperative investigations to enhance the effect of liver function tests. Okamoto[1], Mizumoto[18] and Yamanaka[25] have developed sophisticated score systems including ICG which are able to recognize high risk patients. Yet clinical application is not widely accepted. Although patients with cirrhosis and postoperative complications have lower ICG and GEC values in our study than patients with cirrhosis and uneventful postoperative course this difference is not significant presumably due to the small number of patients.

As the test procedure for determination of GEC is complicated and time consuming the ICG test in our opinion serves best to distinguish high risk patients. The score system proposed by Yamanaka[25], which includes the ICG elimination, is more accurate than the ICG test but it is difficult to perform in daily clinical routine.

Although quantitative liver function tests examined in our study do not correspond to the actual liver volume, they seem to be a valuable parameter to select patients with severe cirrhosis who are likely to suffer from postoperative liver insufficiency. Those patients should be treated by a limited resection.

### REFERENCES

1. Okamoto, E., Kyo, A., Yamanaka, N., Tanaka, N. and Kuwata, K. (1984) Prediction of safe limits of hepatectomy by combined volumetric and functional measurements in patients with impaired hepatic function. *Surgery*, **95**, 586–592.
2. Nagorney, D. M., van Heerden, J. A., Ilstrup, D. M. and Adson, M. A. (1989) Primary hepatic malignancy: Surgical management and determinants of survival. *Surgery*, **106**, 740–747.
3. Takenaka, K., Kanematsu, T., Fukuzawa, K. and Sugimachi, K. (1990) Can hepatic failure after surgery for hepatocellular carcinoma in cirrhotic patients be prevented? *World J. Surg.*, **14**, 123–127.
4. Tsutsuki, T., Sugioka, A., Ueda, M., Iida, S., Kanai, T., Yoshii, H. and Nakayasu, K. (1990) Hepatic resection for hepatocellular carcinoma. *Surgery*, **107**, 511–518.
5. Choi, T. K., Lai Edward, C. S., Fan, S. T., Mok Francis, P. T. and Wong, J. (1990) Results of surgical resection for hepatocellular carcinoma. *Hepato-gastroenterol*, **37**, 172–176.
6. Bismuth, H., Houssin, D., Ornowski, J. and Meriggi, F. (1986) Liver resections in cirrhotic patients: *A western experience. World J. Surg.*, **10**, 311–317.
7. Franco, D., Caspussotti, L., Smadja, C., Bouzari, H., Meakins, J., Kemeny, F., Grange, D. and Dellepiane, M. (1990) Resection of hepatocellular carcinomas; Results in 72 european patients with cirrhosis. *Gastroenerology*, **98**, 733–739.
8. Paquet, K. J., Koussouris, P., Mercado, M. A., Kalk, J. F., Müting, D. and Rambach, W. (1991) Limited hepatic resection for selected cirrhotic patients with hepatocellular or cholangiocellular carcinoma: A prospective study. *Br. J. Surg.*, **78**, 459–465.
9. Lin, T. Y. and Chen, C. C. (1965) Metabolic function and regeneration of cirrhotic and non cirrhotic livers after hepatic lobectomy in man. *Ann. Surg.*, **162**, 959–972.
10. Öllerich, M., Burdelski, M., Lautz, H. U., Schulz, M., Schmidt, F. W. and Herrman, H. (1990) Lidocaine metabolic formation as a measure of liver function in patients with cirrhosis. *Ther. Drug Monit.*, **12**, 219–226.
11. Paumgartner, G. (1975) The handling of ICG by the liver. *Schweizer Med. Wochenschr. Suppl.* 17, **105**, 2–30.
12. Tygstrup, N. (1964) The galactose elimination capacity in control subjects and in patients with cirrhosis of the liver. *Acta Med. Scand.*, **175**, 288–294.
13. Koischwitz, D. (1979) Sonographische Lebervolumenbestimmung: Problematik, Methodik und praktische Bedeutung der

Quantifizierung des Lebervolumens Fortschr. *Röntgenstr.*, **131**, 243–248.

14. Kojima, H. (1978) Relation of the size of functional hepatic cell mass to the clearence of Indocyaninegreen. *Nagoya J. Med. Sci.*, **40**, 47–55.
15. Marchesini, G., Bua, V., Bruhori, A., Branchi, G., Pisi, P., Fabri, A., Zoli, M. and Pisi, E. (1988) Galactose elimination capacity and liver volume in aging man. *Hepatology*, **8**, 1079–1083.
16. Zoli, M., Marchesini, G. and Melli, A. (1986) Evaluation of liver volume and liver function following hepatic resection in man. *Liver*, **6**, 286–291.
17. Nakamura, T. and Nakamura, S. (1961) An approach of measurement of blood flow in intrahepatic shunts in cirrhosis of the liver. *J. Lab. Clin. Med.*, **58**, 455–462.
18. Mizumoto, R., Kawarada, Y. and Noguchi, T. (1979) Pre-operative estimation of operative risk in liver surgery with special reference to functional reserve of the remnant liver following major hepatic resection. *Japanese J. Surg.*, **9**, 343–349.
19. Thomson, A. H., Elliott, H. L. and Kelman, A. W. (1987) The pharmacodynamics and pharmacokinetics of lidocaine and MEGX in healthy subjects. *J. Pharmacokin. Biopharm.*, **15**, 101–107.
20. Ranek, L., Buch-Andreasen, P. and Tygstrup, N. (1976) Galactose elimination capacity as a prognostic index in patients with fulminant liver failure. *Gut.*, **17**, 959–964.
21. Lauterburg, B. H., Sautter, V., Preisig, R. and Bircher, J. (1976) Hepatic functional detoriation after portocaval shunt in the rat. *Gastroenterology*, **71**, 221–227.
22. Lindskov, J. (1982) The quantitative liver function as measured by the galactose elimination capacity. *Acta Med. Scand.* **212**, 295–308.
23. Albers, I., Hartmann, H., Bircher, J. and Creutzfeld, W. (1989) Superiority of the Child-Pugh classification to quantitative liver function tests for assessing prognosis of liver cirrhosis. *Scand. J. Gastroenterol.*, **24**, 269–276.
24. Matsumata, T., Kanematsu, T., Yoshida, Y., Furuta, T., Yanaga, K. and Sugimachi, K. (1987) The Indocyanine green test enables prediction of postoperative complications after hepatic resection. *World J. Surg.*, **11**, 678–681.
25. Yamanaka, N., Okamoto, E., Kuwata, K. and Tanaka, N. (1984) A multiple regression equation for prediction of post-hepatectomy liver failure. *Ann. Surg.*, **200**, 658–663.

